# A Novel Way to Fill Green Gap of GaN-Based LEDs by Pinning Defects in Nanorod Array

**DOI:** 10.3390/nano12213880

**Published:** 2022-11-03

**Authors:** Jinglin Zhan, Zhizhong Chen, Chuhan Deng, Fei Jiao, Xin Xi, Yiyong Chen, Jingxin Nie, Zuojian Pan, Haodong Zhang, Boyan Dong, Xiangning Kang, Qi Wang, Yuzhen Tong, Guoyi Zhang, Bo Shen

**Affiliations:** 1 State Key Laboratory for Artificial Microstructure and Mesoscopic Physics, School of Physics, Peking University, Beijing 100871, China; 2 Inspur (Beijing) Electronic Information Industry Co., Ltd., Beijing 100085, China; 3 State Key Laboratory of High-End Server & Storage Technology, Beijing 100085, China; 4 Dongguan Institute of Optoelectronics, Peking University, Dongguan 523808, China; 5 Yangtze Delta Institute of Optoelectronics, Peking University, Nantong 226000, China; 6 Research Center for Wide Bandgap Semiconductor of PKU, Gao’an 330800, China; 7 State Key Laboratory of Nuclear Physics and Technology, School of Physics, Peking University, Beijing 100871, China

**Keywords:** GaN-based LEDs, green gap, defect-pinning effect, panchromatic and line-scanning CL

## Abstract

Nanorod array and planar green-emission InGaN/GaN multi-quantum well (MQW) LEDs were fabricated by lithography, nano-imprinting, and top–down etching technology. The defect-pinning effect of the nanostructure was found for the first time. The ratio of the bright regions to the global area in the panchromatic CL images of green MQW samples increased from 30% to about 90% after nano-fabrication. The overall luminous performance significantly improved. Throughout temperature-dependent photoluminescence (TDPL) and time-resolved PL (TRPL) measurements, the migration and recombination of carriers in the MQWs of green LEDs were analyzed. It was proved that nanostructures can effectively prevent carriers from being captured by surrounding nonradiative recombination centers. The overall PL integral intensity can be enhanced to above 18 times. A much lower carrier lifetime (decreasing from 91.4 to 40.2 ns) and a higher internal quantum efficiency (IQE) (increasing from 16.9% to 40.7%) were achieved. Some disputes on the defect influence were also discussed and clarified.

## 1. Introduction

The development of gallium-nitride-based (GaN-based) light-emitting-diode (LED) technology has revolutionized modern lighting. Currently, “green gap” is still a significant issue for high-efficiency and high-visual-quality solid-state lighting [1,2]. The peak IQE of LEDs with GaN/InGaN MQWs can reach about 90% in the blue region. Remarkable performance is achieved by AlGaInP-based LEDs in the red region. However, the optical performance of the above two main materials rapidly degrades in the green and yellow regions. The above “gap” in efficiency is known as “green gap”, which limits further advancement in the applications of GaN-based LEDs. The much defective and severely phase-separated (PS) structures [2,3], high polarization field [4], carrier delocalization [5,6], and metallic In precipitates [7] are common in the InGaN active layer with high indium (In) content. This leads to the monotonous reduction of wall-plug efficiencies (WPEs) [8,9]. The pre-strained layers, Si substrate, free-standing GaN substrate, InGaN pseudo-substrate, and patterned sapphire substrates (PSS) are applied to reduce the defects’ density in MQWs [6,8,9,10,11,12,13,14]. Especially, our research group has investigated the method of patterned sapphire substrates (PSS) and reported some new discoveries [15,16]. A high temperature would also be used when the indium can be incorporated [8,10]. The quantum-confined Stark effect (QCSE) is considered to be one of the most dominant causes of green gap [1]. Carriers’ spatial separation due to the polarization field degrades the radiative recombination. Nonpolar and semipolar InGaN LEDs [1,11,17,18], the strain-compensated AlGaN interlayer [19,20], and staggered InGaN QWs [21] are used to optimize the performance of green devices. However, the problem of green gap has not been completely resolved. Furthermore, there are a few post-treatment methods, performed on the grown epitaxial wafer, that were proposed to deal with the defect-related performance degradation. Some discussion has been brought forward in recent years. Some researchers have thought the defects in InGaN layers are not always fatal to luminescence [9,22]. They have found that most defects are passivated in low-In-content material by the potential barriers [22,23]. In order to clarify the above dispute and lay the foundation for future device optimization, the defect-related carrier behaviors in the MQWs of GaN-based green LEDs need to be analyzed in detail.

Stringfellow et al. summarized the microstructures of InGaN epilayers affected by alloy composition, strain, lattice pulling, ordering, phase separation (PS), and Stranski–Krastanov (S–K) growth [24]. InGaN alloy is a non-uniform composition deviated from an ideal, random distribution of the cation species. Its strain and alloy compositions are mutually interdependent [25,26]. Lattice pulling describes the phenomenon between the strain and composition [25]. The PS suppression in 3 nm InGaN QWs is observed when the In content reaches 0.45 [26]. Due to the large lattice mismatch between InGaN and GaN, S–K growth dominates in InGaN/GaN QWs [27]. The transition from 2D to 3D growth occurs at a thickness of about 1.5 nm for In_0.15_Ga_0.85_N. The self-assembled InGaN QDs are formed at the top region of InGaN QWs [28,29]. The edges and apexes of the pyramidal islands elastically relax the strain where In atoms are likely incorporated into InGaN [30]. In some cases, the misfit dislocation production will also cause the plastic relaxation of the island and thus more In incorporation [27]. The free energy of the incoherent island is smaller than that of the coherent one. More rapid growth of incoherent islands can lead to dual wavelength emissions [24]. These incoherent islands become larger when the surrounding small coherent islands coalesce more. The increase of In content may also cause In accumulation in the regions near threading dislocations [31]. When the incoherent islands reach a micro-size, the carrier localization rapidly degrades [5,6]. It is obvious that the coupling factors of defects, strain, PS, and S–K growth should be controlled well to keep the InGaN islands with appropriate sizes and structures for more efficient luminescence in InGaN QWs.

There are also some extrinsic methods to deal with the green gap issue for GaN-based LEDs. Nanostructures and nanomaterials are widely used to optimize the optical performances of the device [32,33,34,35,36,37,38,39,40,41,42,43,44,45]. Wang et al. reported an internal quantum efficiency (IQE) enhancement of 88 times by nanorod array with a 205 nm diameter in green LEDs [32]. A nanocavity effect on green light emission was confirmed. However, the IQE reported in [32] is less than 20% and needs further improvement. The strain relaxation, excitation, and light extraction enhancements have also been considered in nanorod LED arrays [33,34,35,36]. Moreover, the exciton recombination is changed in the nanorods by the interactions with surface states and longitudinal optical (LO) phonons [37,38]. In the previous study of our research group, the effects of nanocavity and photonic crystals in InGaN/GaN nanorod LED arrays were discussed in detail [35]. The other extrinsic method is surface plasmon (SP) [39,40,41]. By embedding Ag nanoparticle (NP) arrays in p-GaN, the photoluminescence (PL) of green LEDs was enhanced by a factor of about 4.5 [39]. The coupling of SP and QWs, which suppress the nonradiative recombination by transferring the energy to a radiative one, leads to the above PL enhancement [40,41]. The nonradiative resonant energy transfer (NRET) between QWs and QDs is another extrinsic method to enhance green light emission [42,43]. The coupled QW–QD nanostructures make the injection and spontaneous emission of green QDs more effective [43]. Furthermore, the introduction of V-pits [22,23], barrier structures, and growth temperature [19,20,46,47] are also extrinsic methods for the improvement of green emissions in QWs. However, there are few clear microstructural images for these extrinsic methods, which hinders the further development of the above technologies.

There are some techniques to characterize the light emissions from InGaN microstructures [5,37,47,48,49,50,51,52,53,54]. Confocal micro-PL (μPL) has been used to obtain emission mapping with the spatial resolution of about 200 nm [5,48,49]. This is slightly larger than the sub-micrometer In-rich cluster. Fluorescence lifetime imaging microcopy (FLIM) shows a similar spatial resolution to the μPL technique to measure green nanorod LEDs [37]. In order to break the diffraction limit, near-field scanning optic microscopy (NSOM) was used [50]. The spatial resolution can reach tens of nanometers. Furthermore, scanning tunneling luminescence (STL) was also reported to obtain a nanometer-scale resolution [51]. A spectral resolution was also observed at 50 meV from a single localized state. STL measurement requires a very thin p-GaN capping layer, which is not convenient to characterize the full structure of green LEDs. Another popular nanoscale luminescent measurement is the cathodoluminescence (CL) technique [47,52,53,54]. In CL mapping, a spectrum is recorded for each position of the electron beam, hence building a hyper-spectral map.

In this study, a green conventional broad LED and nanorod array LED with a diameter of 160 nm were fabricated. The green-emission enhancement of the nanorod was carefully investigated by CL measurements (including panchromatic CL and line-scanning CL measurements). Clear microstructural images of the active regions’ emission performances were achieved. It was found that the micro-sized In-rich cluster was broken into the maximal size of 160 nm in the nanorod array LED. Defects were well-pinned in nanorod arrays. As a result, the overall luminous performance of the nano samples was significantly optimized. Throughout the measurements of temperature-dependence PL (TDPL) and time-resolved PL (TRPL), luminescent mechanisms, especially defect-related migration and recombination processes, were further discussed. Finite difference-of-time domain (FDTD) software was used to assess the excitation and light extraction efficiency (LEE) enhancement of the nanostructure. Compared with previous research [6,8,9,10,11,12,13,14,15,16], a novel post-treatment method to alleviate the defect-related performance degradation of GaN-based LEDs, especially those with high In content, was proposed. Based on the detailed analysis of the carriers’ recombination and migration processes, some disputes [9,22] on the defects’ influence were clarified. A new understanding of the nanostructure’s advantages was also provided. 

## 2. Materials and Methods

InGaN/GaN MQW green LEDs were grown on a 2-inch-patterned sapphire substrate (PSS) with metalorganic chemical vapor deposition (MOCVD). The structure of the epitaxial layer consisted of a 3 μm undoped GaN layer, a 2.5 μm Si-doped n-GaN, 8 periods of InGaN/GaN MQWs with 3 nm wells and 15 nm Si-doped barriers, a 20 nm AlGaN electron barrier layer (EBL), and 160 nm Mg-doped p-GaN. The doping concentrations for n-GaN and p-GaN were 7 × 10^18^/cm^3^ and 1 × 10^20^/cm^3^, respectively. 

The fabrication procedures for the conventional and nano LEDs were as follows: The active regions of the cleaned wafer were defined by photolithography. Conventional LEDs were achieved after inductively coupled plasma (ICP) etching. To fabricate nanorods, a 100 nm SiO_2_ layer was deposited on the surface of the above-achieved conventional LEDs with plasma-enhanced chemical vapor deposition (PECVD). The pattern of the nano-arrays was transferred from a stamp to the SiO_2_ layer with nanoimprint lithography (NIL). An SiO_2_ layer was used as a mask during the second ICP etching. After that, GaN-based nano LEDs were completely prepared. The size of the conventional LED chips were 550 × 220 μm^2^. The top diameter, bottom diameter, and height of the nanorods were about 160, 417, and 400 nm, respectively. 

**Figure 1:**
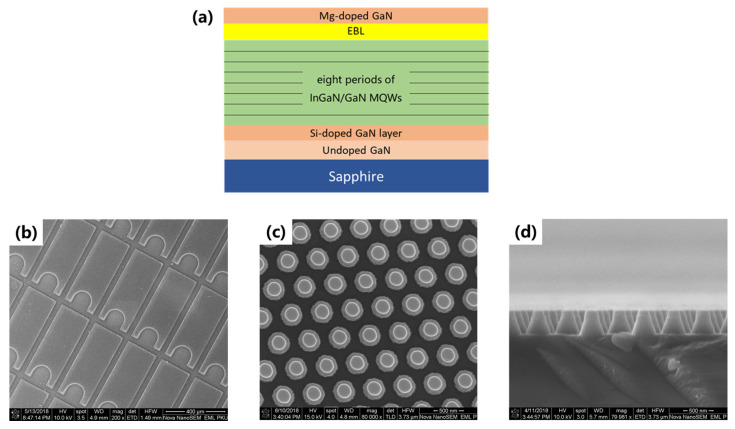
(**a**) The structure of InGaN/GaN MQW green LEDs; (**b**) top-view SEM image of conventional LEDs; (**c**) top-view SEM image of nanorod-array LEDs; (**d**) side-view SEM image of nanorod array LEDs.

Scanning electron microscope (SEM) images of the conventional and nano samples are shown in Figure 1. CL measurements were performed on a Gatan MonoCL4 system at room temperature. A 375 nm pulsed laser with a 69 ps pulse width and 1 MHz repletion rate was used for TRPL measurements. By using a liquid nitrogen temperature control system, PL decay curves under 77 K were achieved. Throughout the TDPL measurements, samples were mounted in a closed-cycle helium cryostat and excited by a steady-state 405 nm laser. The ambient temperature could be changed from 10 to 300 K. The excitation and light extraction of the LED samples were also simulated using FDTD software (FDTD Solutions 2020a, Vancouver, BC, Canada).

## 3. Results

**Figure 2:**
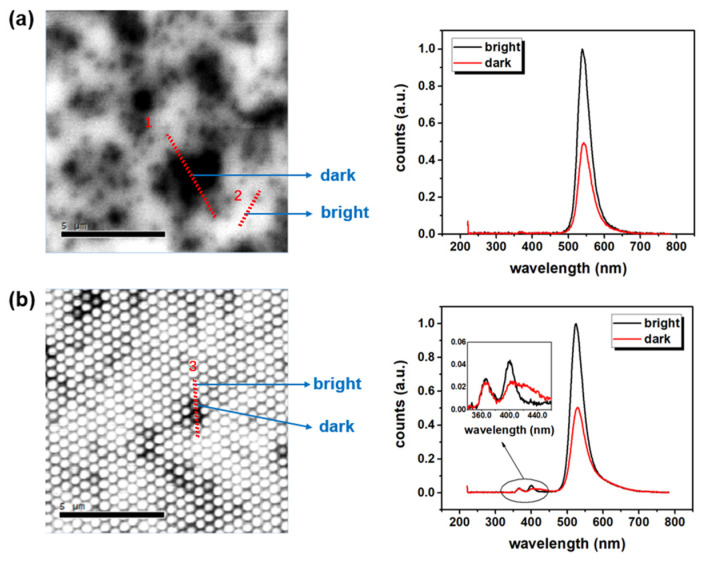
(**a**) Panchromatic CL image of conventional LED and two spectra at marked bright and dark regions; (**b**) CL panchromatic image of nanorod array LED and two spectra at marked bright and dark regions. The inset shows the magnified CL spectra in the range of 350–450 nm.

Figure 2 shows the panchromatic CL images of the conventional and nanorod array LEDs and their corresponding CL spectra in bright and dark regions. There are many dark regions in Figure 2a, including many small points with the size of tens of nanometers and a few large regions with a size on the micrometer scale. The obvious shadows were observed near the large dark region and high-density small-point region. Therefore, the ratio of the bright regions to the global area was smaller than 30%. In the CL spectra, the peak intensity in the bright region was two times of that in the dark region. The peak wavelength blueshift was about 3.1 nm, and the spectral width was narrowed from 45.8 to 42.7 nm. Contrary to the conventional LED, there were no micron-sized dark regions in the CL panchromatic image of the nanorod array LED, as shown in Figure 2b. A few dark nanorods were scattered across the whole image. The ratio of the bright region reached about 90% in the panchromatic CL image. In the CL spectra, the green emission peak intensity, wavelength, and spectral width changed from bright to dark, similar to that in the conventional LED. A blue shift of about 13.5 nm occurs in nanorods, which can be attributed to an approximately 30–40% strain relaxation and the effective alleviation of QCSE in QWs [55]. Two peaks, with the wavelengths of 365.3 and 399.6 nm, existed in the spectra detected from the bright region. In the dark region, at the wavelength of 400 nm, the peak intensity decreased to half, and the spectral width broadened to about twice of those in the bright region.

The dark regions always showed a low intensity, long wavelength, and large spectral width compared with the neighbored bright regions, whether they were located in the conventional or nanorod samples, or small or big regions. Because the strain and composition of InGaN QWs are coupled in most cases [24], both of these may cause a long wavelength emission. As reported previously, the QD-like In-rich clusters showed a high intensity and shallow spectral width [43,51], while the micro-sized In-rich cluster caused a low intensity and large spectral width by defects and/or carrier delocalization [2,5]. As seen in Figure 2b, the large dark regions were broken by nanorod arrays and only one or no neighbored nanorods were affected. This indicated that the dark regions were mainly caused by defects of the nano samples. These defects included threading and misfit dislocations, which could have also enhanced the In incorporation [27,31]. In the planar one, the micro-sized clusters might be due to carrier delocalization, strain, and defects, while the large blueshift of 13.5 nm in Figure 2b showed the effective strain relaxation of nano-arrays [33,34]. Apart from the green emissions, the CL spectra of nanorod array LEDs also showed two peaks at 365 and 400 nm. These peaks corresponded to the recombination of the GaN band-edge and InGaN shallow wells. These excessive peaks appeared clearly when the epi-structures were etched down to n-GaN. Furthermore, the threading dislocations (TDs) in the dark region also affected the shallow well’s growth, which caused inhomogeneity and more In incorporation [31].

**Figure 3:**
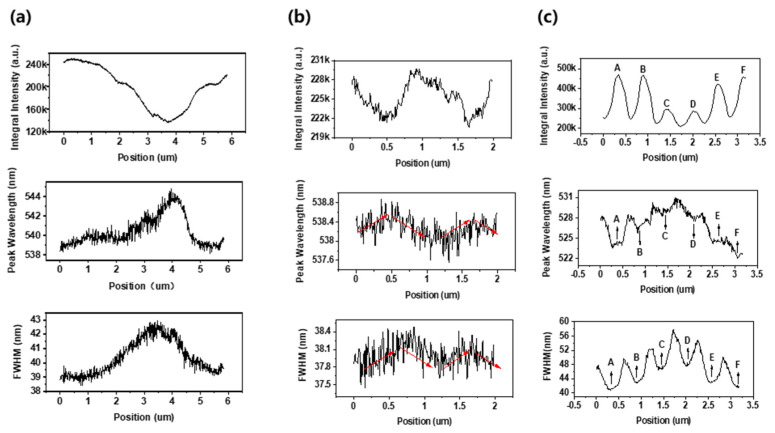
Integral intensity, peak wavelength, and FWHM of green emissions in CL line-scanning along: (**a**) red line 1 through dark region, (**b**) red line 2 through bright region in Figure 2a, (**c**) red line 3 though two dark nanorods in Figure 2b. Six nanorod centers are marked with the letters A–F.

The panchromatic CL images did not give clear pictures in the dark and bright regions. CL line-scanning provided the emission details in these regions. Figure 3 shows the CL scanning results along red lines 1, 2, and 3 in Figure 2, which penetrated through the dark and bright regions. The scanning step was 10 nm. The integral intensity, peak wavelength, and full width at half maximum (FWHM), which were fitted by the Gaussian function, were extracted from the CL spectra. From the edge to the center in the dark region, the intensity decreased, and the wavelength and FWHM gradually increased, as shown in Figure 3a. The extremes were roughly located at the center of the dark region, which did not strictly overlap. There were some terraces or shoulder peaks in these curves. Considering the carrier diffusion, the spatial resolution of the CL measurement was about 50 nm [53]. Therefore, it can be concluded that the several-microns dark region included different sub-regions with a different In content and/or strain status. These sub-regions might have formed by a coalescence of the islands, which were either coherent or incoherent [24]. The other production method was spinodal decomposition by strain relaxation, which may have been elastic or plastic relaxation [27,30]. There did not seem to be any potential barriers between these islands, where the carriers could diffuse to the defective center region. Subsequently, even the coherent islands in the dark region showed low CL intensity. Figure 3b shows the CL scanning results in the bright region. Line 2 passed through two dark points. A low intensity, long wavelength, and high FWHM were also observed. The integral intensity fluctuated by less than 4%, and the peak wavelength and FWHM deviated by about 0.4 nm along red line 2. These figures were much smaller than those in the dark regions of Figure 3a. As a result, these dark points can be attributed to some isolated defects. Considering the small size and slight potential energy difference, the probability of carriers being captured by these defective center regions was not very high [22]. The dark points shown in Figure 3b played a minor role in the overall CL of the samples.

Figure 3c shows the CL line-scanning results of the nanorod array LED. The nanorod centers are marked with the letters A–F. The CL scanning results showed the fluctuation properties according to the period of the nanorod array. From the profiles of the three curves, it can be deduced that there was a micro-sized In-rich region along red line 3 before the nanorod arrays were fabricated. For each nanorod, the integral intensity distribution was Gaussian-like. This was because the pillar side was less excited and more defective than the pillar center through electron impinging. The nanorod strain relaxation caused a blueshift of 13–15 nm of the peak wavelength to the planar LEDs. According to [33], the strain relaxation was about 30%. The peak wavelength curves in the bright nanorods were almost linear, while they were complex in the dark nanorods. This meant that lattice latching occurred in the bright nanorods, and defects dominated in the dark nanorods [24]. The FWHMs of the nanorods were 2–5 nm larger than those of the planar ones. This was due to yellow-band luminescence when n-GaN was exposed by etching. The yellow band overlapped with the green emission from the InGaN QWs. In the streets between the nanorods, the yellow band became more significant, which led to a longer peak wavelength and higher FWHM. It was observed that the bright nanorods showed different peak wavelengths and FWHMs. This indicated that high-In content clusters may efficiently emit light when the carriers are prevented from the nonradiative center of defects [22]. These high-In content clusters in bright nanorods might have been formed by coherent growth or elastic strain relaxation, which produced a low-defect density [30]. Here, we assumed that the strain difference was small between the neighbored nanorods. The dark nanorods C and D showed half intensity, a longer wavelength, and a higher FWHM compared with those of the bright nanorods. This meant that the two dark nanorods should have high-density nonradiative recombination centers, such as TDs or misfit dislocations.

**Figure 4:**
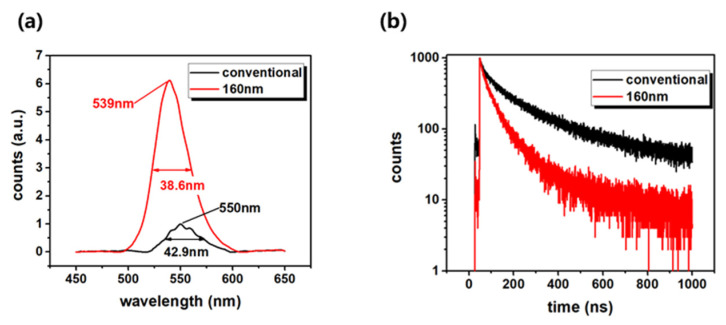
(**a**) PL spectra at room temperature, (**b**) TRPL spectra at 77 K of conventional and nano samples.

The PL spectra of the conventional and nano samples were measured at room temperature, as shown in Figure 4a. The PL was excited by a laser with a wavelength of 405 nm and spot size of 2 mm. Different from ultra-high-resolution CL measurements, the PL spectra showed an overall optical performance within several millimeters of the conventional and nano samples. Surprisingly, the integral PL intensity of the nanorod LED was 6.2 times higher than that of the conventional one. Considering the reduced active regions, the integral PL power density of the nanorod LED was 18.6 times higher than that of the conventional one. The peak wavelengths were 539 and 550 nm for the nanorod and conventional LEDs, respectively. The FWHMs were 38.6 and 42.9 nm for the two samples, respectively. These results were similar to the CL results. The luminescence differences were due to the excitation energy and volume of the PL and CL measurements. The kiloelectronvolt energy of CL can excite all the transitions in LEDs, while 405 nm lasers only excite the transitions in QWs. With the CL measurement, the exposure of n-GaN also affected the CL spectra. The PL measurements were performed in a large area, which showed the average macro results. The PL intensity enhancement of the nanorod LED can be attributed to the improvements of IQE, LEE, and excitation efficiency [32,35], which will be discussed later. The FWHM of the nanorod LED was less than that of the planar one. If the yellow-band luminescence of n-GaN and the sidewall are neglected, it is reasonable to expect a small FWHM, because the strain is well-relaxed in nanorods [33].

Figure 4b shows the TRPL spectra measured in the conventional and nanorod LEDs at the temperature of 77 K. Based on the stretched exponential model [56,57], PL intensity *I*(*t*) decay can be represented as:

(1) $$I\left(t\right)=I_{0}\exp\left(-{\left(\frac{t}{\tau }\right)}^{\beta }\right)$$

where *I*_0_ represents the initial intensity, β represents the localization state distribution, and τ is the initial lifetime. β ranges from 0 to 1; β=1 means a single-exponential decay. According to the fitting results of the carrier decay curve shown in Figure 4b, the τ of the nanorod LED was 40.2 ns, which was much faster than the 91.4 ns time of the conventional LED. The nanorod’s effective strain relaxation and nanocavity effect resulted in higher radiative recombination rates [33,34,35]. The β of the nanorod LED was 0.72, which was larger than the 0.55 one of the conventional LED. The larger β can also be attributed to less extension of the localization state [56]. As shown in Figure 2, 90% bright nanorods were insulated to the nonradiative recombination centers in the dark nanorods.

**Figure 5:**
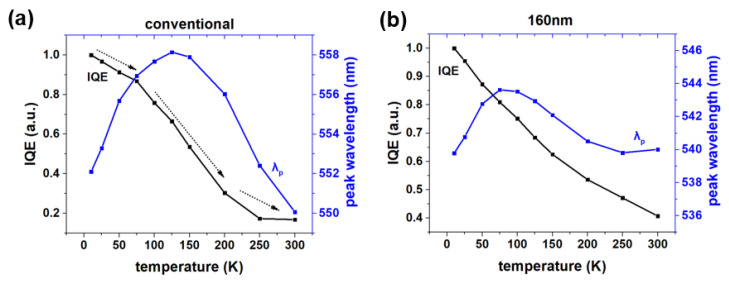
IQE and peak wavelength dependence on temperature drawn from TDPL spectra for (**a**) conventional and (**b**) nanorod LEDs. (Black line: IQE changes with the increase of temperature; blue line: peak wavelength changes with the increase of temperature.)

TDPL measurements were used to determine the IQE and carrier localization [58,59]. Assuming the nonradiative recombination was almost inactive at 10 K, the IQE measured at different temperatures can be calculated by the following formula [58]:

(2) $$IQE_{T}=\frac{I_{T}}{I_{10K}}$$

where *I_T_* and *I*_10*K*_ are the PL integral intensity measured at the temperatures of T and 10 K, respectively. The IQEs at 300 K for the conventional and nano LEDs were 16.9% and 40.7%, respectively, as shown in Figure 5. In Figure 2a, there is shown large amounts of micro-sized In-rich clusters, where the long peak wavelength indicated the local potential minima. Enormous defects and severe polarization fields in these regions were detrimental to the LED’s radiative recombination processes. As a result, the IQE of conventional samples quickly dropped in the temperature range of about 100 to 200 K, when carriers were gathered in or around micro-sized In segregation regions. Due to the defects’ pinning effects and the strain relaxation and transverse restrictions of the carrier migration in the nanostructures, the IQE of the green LEDs was greatly improved. On the other hand, the S-shaped shifts of temperature-dependent peak wavelengths were considered to be the results of the carriers’ migration in and out of the local potential minima [59]. For the conventional LED, redshift occurred when the temperature increased from 10 to about 125 K, which corresponded to the frozen carriers transferring into the local potential minima. After 125 K, the occupation of higher-energy levels in localized states and carrier delocalization processes led to blueshift. Due to the large size of the localization region, the second redshift could not appear in the conventional LED [49]. For the nano LED, the transition temperature of peak wavelengths from redshift to blueshift was 75 K, which was less than 125 K. There was a 3.8 nm redshift for the nanorod LED, which was less than the 6.0 nm redshift of the conventional LED. The above phenomena were caused by breaking the large micro-sized In-rich clusters by nanorod arrays. Carriers were confined to single nanorods in which potential fluctuations were not significant. After 250 K, the process of carriers overflowing from low potential energy still existed, which led to a blueshift tendency. However, thermal-caused bandgap shrinkage became serious [59]. The redshift caused by thermal Eg shrinkage exceeded the blueshift caused by the carrier delocalization processes. As a result, there was an overall redshift tendency for the nanorods when the temperature was higher than 250 K.

**Figure 6:**
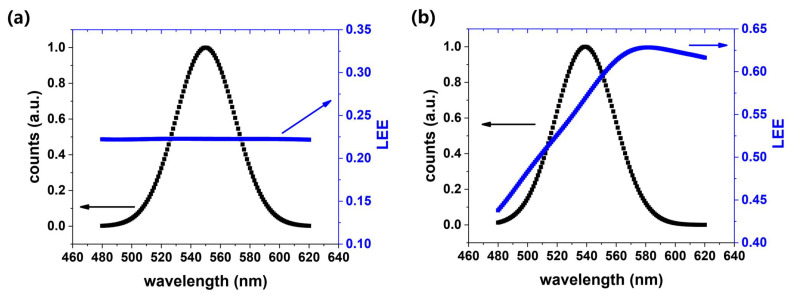
FDTD simulation results of LEE (blue line) and spectra given by Gaussian approximation (black line): (**a**) conventional (**b**) nanorod LEDs.

FDTD simulation was used to assess the LEEs of the conventional and nanorod LEDs. According to the previous studies [34,35], electron-hole radiative recombination can be simplified as a dipole, which was placed in the middle of the MQWs. The spectrum of the dipole source had a Gaussian shape. The peak wavelengths of the conventional and nano LEDs were set as 550 and 539 nm, respectively. The FWHM of 40 nm was used throughout the FDTD simulation. The overall LEE of the device can be obtained by the weighted average of the LEE at different wavelengths:

(3) $$\eta_{LEE}=\frac{\int \eta_{LEE}\left(\lambda \right)\times I\left(\lambda \right)d\lambda }{\int I\left(\lambda \right)d\lambda }$$

where $\eta_{LEE}\left(\lambda \right)$ is the LEE at the specific wavelength *λ*. The corresponding spectral intensity is represented as I(λ). According to the results of the simulation which are shown in Figure 6, the overall LEE of the conventional and nano samples were calculated as 22.2% and 56.7%, respectively. As is shown in Figure 6, the LEE of the conventional LED kept stable in the wavelength range from 480 to 620 nm. However, there was an obvious change of LEE for nanorods, which can be attributed to the nanocavity effect [32,35]. When the light emission at a certain wavelength resonates with the nano-resonant cavity, the LEE of nanorods can be greatly enhanced. As seen in Figure 6b, the resonant wavelength of our nano samples, which was located at the peak of the LEE, was around 580 nm.

Based on the above analysis, the PL intensity enhancement shown in Figure 4a can be well explained. Considering the reduced active regions, the integral PL power density of the nanorod LED was 18.6 times higher than that of the conventional one. After nano-fabrication, the enhancements of IQE and LEE reached to 2.41 and 2.58 times, respectively. The laser excitation efficiency enhancement of the nanorods was reported to be about three times [40]. Considering the above three factors, the PL integral intensity of the nano LED can be enhanced to above 18 times, which was consistent with the results of the PL measurement.

According to the discussion of this paper, the nanorods’ extraordinary performance can be attributed to strain relaxation, defect-pinning effects, and nanocavity effects. However, the role ratios of the above three effects are still not clear and need further study. Furthermore, in order to completely solve the green gap issue, research on the intrinsic methods, which consider the couple influence of strain, composition, phase separation, and S–K growth, also need to be conducted in the future.

## 4. Conclusions

Nanorod array and planar green-emission InGaN/GaN multi-quantum well (MQW) LEDs were fabricated by photolithography, nano-imprinting, and ICP techniques. Micro-structural images of the active regions’ emission performances were detected by high-resolution panchromatic CL and line-scanning CL measurements. The ratio of the bright regions to the global area in the MQWs of planar green LEDs was smaller than 30%, which increased to about 90% for the nanorods. The defect-pinning effect in the nanorods was found for the first time, which deepens the traditional understanding of the advantages of nanostructures. Throughout the measurements of TRPL and TDPL, the migration and recombination of carriers in the MQWs of green LEDs were analyzed in detail. Less extension of the localization state was verified. Carriers in the MQWs of nanorods could hardly be captured by surrounding nonradiative recombination centers, which enhanced and accelerated the luminescence processes. The PL power density of the nano LED was 18.6 times higher than that of the conventional one. The carrier lifetime of the nanorod LED was 40.2 ns, which was much faster than the 91.4 ns one of the conventional LED. The IQE and LEE of nanorod LEDs were improved by 2.41 and 2.55 times, respectively. Considering the laser excitation enhancement, the PL enhancement can be well explained. According to the previous discussions, a novel post-treatment method to deal with the performance degradation caused by defects was proposed, which was very effective for the optimization of GaN-based MQW devices, especially those with high In content.

## Data Availability

The data presented in this study are available upon request from the corresponding author.
